# Defining new criteria for selection of cell-based intestinal models using publicly available databases

**DOI:** 10.1186/1471-2164-13-274

**Published:** 2012-06-22

**Authors:** Jon Christensen, Sara El-Gebali, Manuela Natoli, Thierry Sengstag, Mauro Delorenzi, Susanne Bentz, Hanifa Bouzourene, Martin Rumbo, Armando Felsani, Sanna Siissalo, Jouni Hirvonen, Maya R Vila, Piercarlo Saletti, Michel Aguet, Pascale Anderle

**Affiliations:** 1 Institute for Macromolecular Chemistry and Center for Biological Signaling Studies (BIOSS), University of Freiburg, Freiburg, Germany; 2 Institute of Biochemistry and Molecular Medicine, University of Berne, Berne, Switzerland; 3 Istituto di Biologia Cellulare e Neurobiologia, CNR, Rome, Italy; 4 OSC - Omics Science Center, RIKEN Yokohama Institute, Yokohama, Japan; 5 Swiss Institute of Bioinformatics, Lausanne, Switzerland; 6 Swiss National Centre of Competence in Research Molecular Oncology, Swiss Institute for Experimental Cancer Research, Ecole Polytechnique Fédérale de Lausanne, School of Life Sciences, Lausanne, Switzerland; 7 NCCR TransCure, University of Berne, Berne, Switzerland; 8 Institute of pathology of Lausanne, Centre hospitalier et universitaire vaudois, Lausanne, Switzerland; 9 Laboratorio de Investigaciones del Sistema Inmune (LISIN), Facultad de Ciencias Exactas, Universidad Nacional de La Plata, La Plata, Argentina; 10 Faculty of Pharmacy, University of Helsinki, Helsinki, Finland; 11 LEITAT Technological Center, Barcelona, Spain; 12 Medical Oncology Department, IOSI, Ospedale Regionale di Lugano, Lugano, Switzerland; 13 Swiss Institute for Experimental Cancer Research, Ecole Polytechnique Fédérale de Lausanne, School of Life Sciences, Lausanne, Switzerland; 14 Institute of Biochemistry and Molecular Medicine, University of Bern, Buehlstrasse 28, 3000, Bern 9, Switzerland

**Keywords:** Cell lines, Genomic profiling, Malignant traits, Epithelial-mesenchymal transition, Intestine, Colon cancer, Chemosensitivity

## Abstract

**Background:**

The criteria for choosing relevant cell lines among a vast panel of available intestinal-derived lines exhibiting a wide range of functional properties are still ill-defined. The objective of this study was, therefore, to establish objective criteria for choosing relevant cell lines to assess their appropriateness as tumor models as well as for drug absorption studies.

**Results:**

We made use of publicly available expression signatures and cell based functional assays to delineate differences between various intestinal colon carcinoma cell lines and normal intestinal epithelium. We have compared a panel of intestinal cell lines with patient-derived normal and tumor epithelium and classified them according to traits relating to oncogenic pathway activity, epithelial-mesenchymal transition (EMT) and stemness, migratory properties, proliferative activity, transporter expression profiles and chemosensitivity. For example, SW480 represent an EMT-high, migratory phenotype and scored highest in terms of signatures associated to worse overall survival and higher risk of recurrence based on patient derived databases. On the other hand, differentiated HT29 and T84 cells showed gene expression patterns closest to tumor bulk derived cells. Regarding drug absorption, we confirmed that differentiated Caco-2 cells are the model of choice for active uptake studies in the small intestine. Regarding chemosensitivity we were unable to confirm a recently proposed association of chemo-resistance with EMT traits. However, a novel signature was identified through mining of NCI60 GI50 values that allowed to rank the panel of intestinal cell lines according to their drug responsiveness to commonly used chemotherapeutics.

**Conclusions:**

This study presents a straightforward strategy to exploit publicly available gene expression data to guide the choice of cell-based models. While this approach does not overcome the major limitations of such models, introducing a rank order of selected features may allow selecting model cell lines that are more adapted and pertinent to the addressed biological question.

## Background

A wide panel of intestinal cell lines is being used to study the biology of the intestine. All of these cell lines are either directly derived from primary colo-rectal cancers (CRCs) of different clinical stages and differentiation grades or from metastatic sites originated from a colon tumor. The major oncogenic pathways in colon cancer include loss of function mutations in APC, TP53 and SMAD4 (approximately 80–85% of sporadic tumors), or DNA mismatch repair genes, and activating mutations in beta-catenin [1]. As a consequence, the Wnt pathway is activated in most tumors and derived CRC cell lines, albeit to a different extent, depending on the genetic lesions [2-5]. The morphology, expression of differentiation markers, migratory characteristics and their potential to form metastases differ vastly between the cell lines [5-8].

While cancer drug discovery has mainly focused on targeting tumor cell proliferation, the outcome of a cancer depends largely on tumor invasion and dissemination [9]. Recent advances in understanding underlying mechanisms in cancer biology including cancer stem cell (CSC) properties and epithelial-mesenchymal transition (EMT) and their relation to drug susceptibility require that relevant traits are considered for choosing appropriate cell-based models. It is believed that at the invasive front, the tumor cells undergo EMT resulting in increased migratory capacity. Furthermore, EMT has recently been linked in breast cancer to stem cell like properties [10] as well as resistance to chemotherapy in different tumor types including CRC [11-14].

The variety of available human cancer cell lines reflects the genomic heterogeneity across the human cancer population at least in part and has therefore regained attention notably to predict responsiveness of anticancer drugs [15,16]. Starting with the landmark paper by Scherf et al. [17] numerous studies followed that aimed at linking drug response in terms of growth inhibition with gene expression signatures, some specifically focusing on colon cancer (e.g. [18-26]). To our knowledge, no study has specifically focused on linking expression of malignant traits (i.e. EMT, WNT activity, stemness signatures) in colon cancer cell lines to response to therapy.

In this study we compare for the first time gene expression signatures relating to a wide panel of commonly known intestinal cell lines, primary cell cultures of human cancer-associated fibroblasts and laser-dissected human colonocytes, small intestinal enterocytes and tumor cells. We delineate selection criteria for CRC derived cell lines based on genomic expression patterns related to clinical parameters, migratory capacities and proliferative activities. While some cell lines are mainly being used to study mechanisms associated with tumor biology others serve as models for normal enterocytes studying drug absorption. Thus, we used the same strategy to assess the validity of models for oral drug absorption.

## Results

Most dominant gene expression differences as revealed by principal component analysis (PCA) were observed between normal epithelium and all tumor-derived cell lines, and cells with epithelial versus mesenchymal properties.

**Figure 1:**
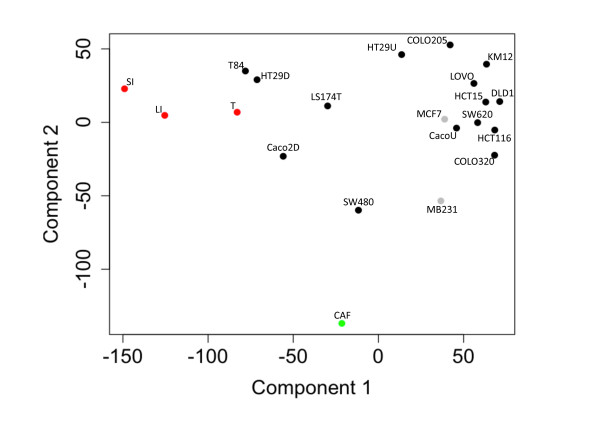
**Most dominant gene expression differences between normal epithelium and all tumor-derived cell lines, and cells with epithelial versus mesenchymal properties.** Principal component analysis (PCA) of genomic expression data from human colon carcinoma cell lines (black); laser-dissected tissues (red) of normal human colonocytes (LI), tumor cells (T) and small-intestinal enterocytes (SI); primary cell cultures (green, CAFs); breast cancer cell lines (grey). For each sample 2–3 three replicates were measured.

PCA was performed to assess the major gene expression differences between microdissected normal and tumor epithelium, primary cancer-associated fibroblast (CAF) cultures and the various CRC cell lines. PCA involves a mathematical procedure that transforms a number of possibly correlated variables into a smaller number of uncorrelated variables called principal components. Hence, the first principal component accounts for as much of the variability in the data as possible and each succeeding component accounts for as much of the remaining variability as possible. Two breast cancer cell lines with known pronounced (MB231) or weak (MCF7) EMT traits [27] were included into the comparison. The first component was defined mainly by the difference between small intestinal enterocytes and all CRC cell lines grown to subconfluency, while the second component was delineated by the difference between CAFs and epithelial cells (Figure 1). T84, HT29 and Caco-2 cells grown to confluency for 3 weeks seemed to be most similar to the small intestinal enterocytes, and also to laser-dissected tumor cells, while SW480 and MB231 proved closest to CAFs.

### Selection of relevant cell lines to study tumor biology based on expression of signatures related to malignant traits

**Figure 2:**
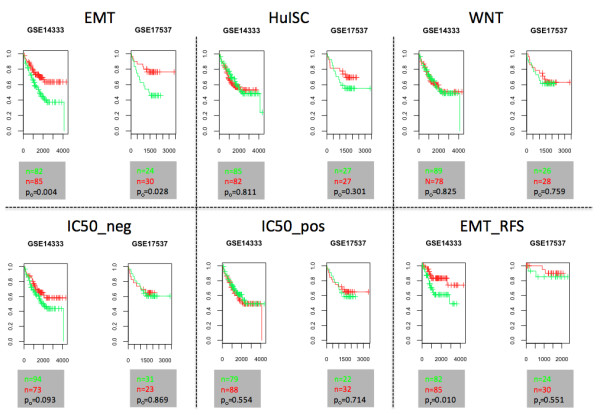
**Signatures predictive for survival.** Kaplan-Meier representation of overall survival and recurrence-free survival (EMT_RFS) probability over time for patients bearing colon cancers with average high (i.e. relative average expression > 0, green) or low expression (i.e. relative average expression < 0, red) of a selection of gene sets (details see Table 1).

Next, the suitability of the various cell lines for tumor biology related studies was assessed. There are no standard models and selection criteria are not well defined. The activation of the Wnt pathway is a hallmark in colon carcinogenesis. Recent findings indicate that the Wnt activity defines CRC stem cells and is regulated by the microenvironment [28]. Thus, the overall Wnt signaling activity was compared in the various samples. When comparing the average expression of direct Wnt targets (i.e. 24 Wnt target genes relevant to human colon and/or other cancers, with a proven direct transcriptional control through TCF binding sites as described in [29]), the SW620 cell line showed the strongest Wnt signal followed by SW480, laser-dissected tumor cells, COLO320, DLD1 and undifferentiated Caco-2 cells (Table 1, detailed description of all gene sets see supplementary data section “expression data analysis” and Additional file 1: Table S2). The ranking of cell lines with strong, medium and low average expression of direct Wnt targets was in agreement with the Wnt activity based on TOPFLASH assays reported by Rosin-Arbesfeld and co-workers [4]. Moreover, the expression of genes involved in EMT ([29]) and a set of genes, recently being described as “humanized intestinal stem cell signature” (HuISC), which identified recurrent CRC [30], were studied to rank the cell lines as models for tumor cells at the invasive front and/or tumor cells with stemness characteristics. Survival analysis studies showed that risk of recurrence was significantly higher for CRC patients with a strong EMT signature and also correlated with overall survival (Figure 2). As expected CAFs and MB231 (positive controls) had the strongest expression of the EMT signature followed by COLO320, SW480, SW620 and laser-dissected tumor epithelial cells, normal colonocytes and differentiated Caco-2 cells. Though COLO320 cells ranked highest of all intestinal epithelial cells, they also had the highest expression of CDX2, a key player in the maturation of intestinal enterocytes [31]. When the cell lines were ranked to the overall expression of stem cell related genes (HuISC), the five top ranked samples were CAF > MB231 > laser-dissected tumor cells > SW480 > LOVO (Table 1). In summary, with respect to the Wnt, EMT and HuISC signatures, SW480 proved the cell line with the strongest traits and a gene expression pattern most similar to that of micro-dissected tumor epithelium.

**Table 1:** Rank order of cell lines according to their signature strength

**Cell Line**	**EMT**	**HuISC**	**WNT**	**Trans_BC**	**SLC_SI3**	**SLC_PM**	**ABC**	**MKI67_CO**	**IC50_pos**	**IC50_neg**
CAF	1	1	8	29	24	23	30	24	6	7
MB231	2	2	23	30	30	29	27	10	10	21
COLO320	3	11	4	17	27	28	10	4	4	29
SW480	4	4	2	28	25	27	17	17	24	12
SW620	5	9	1	27	29	22	20	8	7	26
Tumor	6	3	3	20	8	24	26	23	5	20
Normal	7	10	26	12	7	14	9	27	2	18
Caco,Fl,D	8	15	14	6	6	5	6	21	8	13
Caco,Fl,U	9	7	6	5	11	6	2	13	16	1
Caco_CDX2,U	10	6	21	7	13	7	7	18	11	23
Caco_CDX2,D	11	25	25	3	3	3	4	28	29	14
JE	12	29	30	13	4	12	22	29	3	30
KM12	13	22	20	10	23	11	21	2	13	5
HCT116	14	12	24	21	26	25	18	14	9	24
Caco,Fi,D	15	26	27	2	2	2	5	26	17	19
T84	16	17	18	26	9	17	29	22	22	25
CacoReady	17	19	16	4	5	4	1	20	15	10
HT29,R	18	16	11	24	16	10	13	5	25	3
DLD1	19	13	5	9	18	15	24	3	18	2
Caco,S	20	27	15	23	22	20	11	9	28	6
HT29,S	21	18	13	18	15	13	12	1	20	4
MCF7	22	24	28	25	28	30	25	15	14	28
HT29,D	23	28	22	22	10	21	16	25	26	8
Caco,R	24	14	12	16	19	18	8	7	27	11
IL	25	30	29	1	1	1	3	30	1	27
HT29,U	26	23	19	19	14	9	23	16	30	17
LOVO	27	5	7	14	21	16	19	6	12	15
LS174T	28	8	10	15	12	26	28	19	23	16
COLO205	29	20	9	11	17	8	15	12	21	22
HCT15	30	21	17	8	20	19	14	11	19	9

EMT = EMT-related genes, HuISC=“Humanized intestinal stem cell signature”, WNT = direct Wnt targets, Trans_BC = Transporters known to play important role in oral drug absorption, SLC_SI3 = solute carriers expressed at a significantly higher level in small intestinal samples versus colon samples, SLC_PM = solute carriers of the plasma membrane, ABC = ABC transporters, MKI67_CO: Set of genes correlating with MKI67 expression in colon cancer samples, IC50_pos, IC50_neg: Genes with either strong positive or negative correlation between GI50 values of chemotherapeutics and expression across 60 cell line panel (for details of signatures see Additional file 6, methods). Tumor = laser dissected tumor cells, Normal = laser dissected colonocytes, JE, IL = laser dissected enterocytes of the jejunum or ileum, respectively, D = differentiated, U = undifferentiated, Fi = grown on filters, Fl = grown in flasks, R = resistant to methotraxate, S = sensitive to methotrexate.

### Gain of malignant traits and loss of differentiation signature were associated with migratory properties

**Figure 3:**
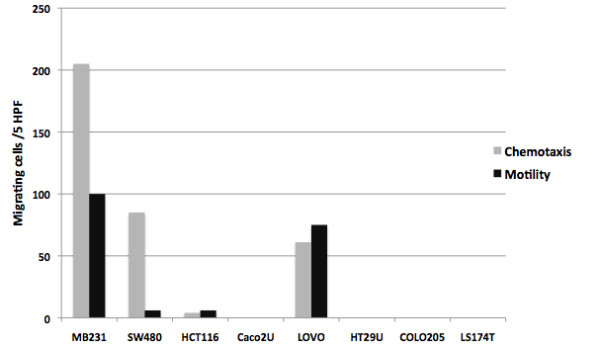
**Gain of malignant traits and loss of differentiation signature are associated with migratory properties.** Migratory properties of intestinal cell lines across membrane in the absence (motility) or presence (chemotaxis) of a FCS gradient (details see “Material and Methods” section). Cell lines are ordered in dependence of rank orders of EMT and SLC_SI3 signatures as shown in Table 1.

Invasive tumor cells acquire higher motility through EMT [9]. Thus, we elucidated to what extent genomic expression characteristics could be linked to migratory properties. In general, the analysis revealed that higher migratory propensity was associated with strong EMT traits and weak expression of a signature derived from normal small intestinal epithelium (i.e. SLC_SI3). Accordingly, SW480 and MB231 cells showed the highest migratory capacity (Figure 3).

### Transporter profile defines absorption model

The model of choice to evaluate drug absorption are Caco-2 cells as they undergo in vitro spontaneous enterocytic differentiation by developing an apical brush border membrane endowed with hydrolases such as sucrase-isomaltase and aminopeptidase N [7,32-36]. On the other hand, comparison of permeability coefficients across different laboratories is still an issue [37]. Various studies have compared the genomic profiles of Caco-2 cells to large and small intestinal tissue [5,38-40]. None of these has systematically compared these profiles to the ones of small intestinal enterocytes in comparison to other intestinal cell lines, and, in particular, to profiles of the genes relevant for active transport.

**Figure 4:**
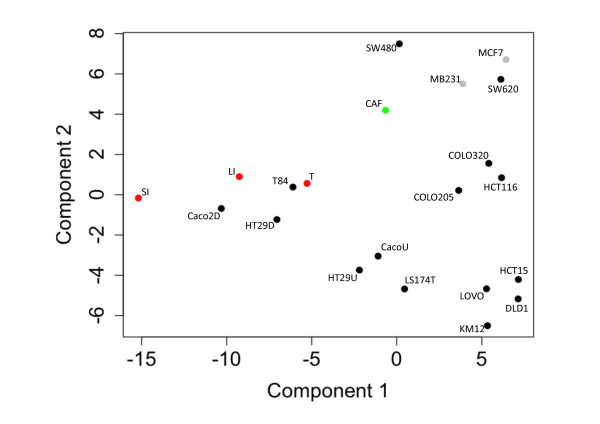
**Caco-2 cells most similar to small intestinal enterocytes with respect to expression of solute carriers.** Principal component analysis (PCA) as shown in Figure 1, but carried out on data set filtered using probe sets representing solute carriers.

**Figure 5:**
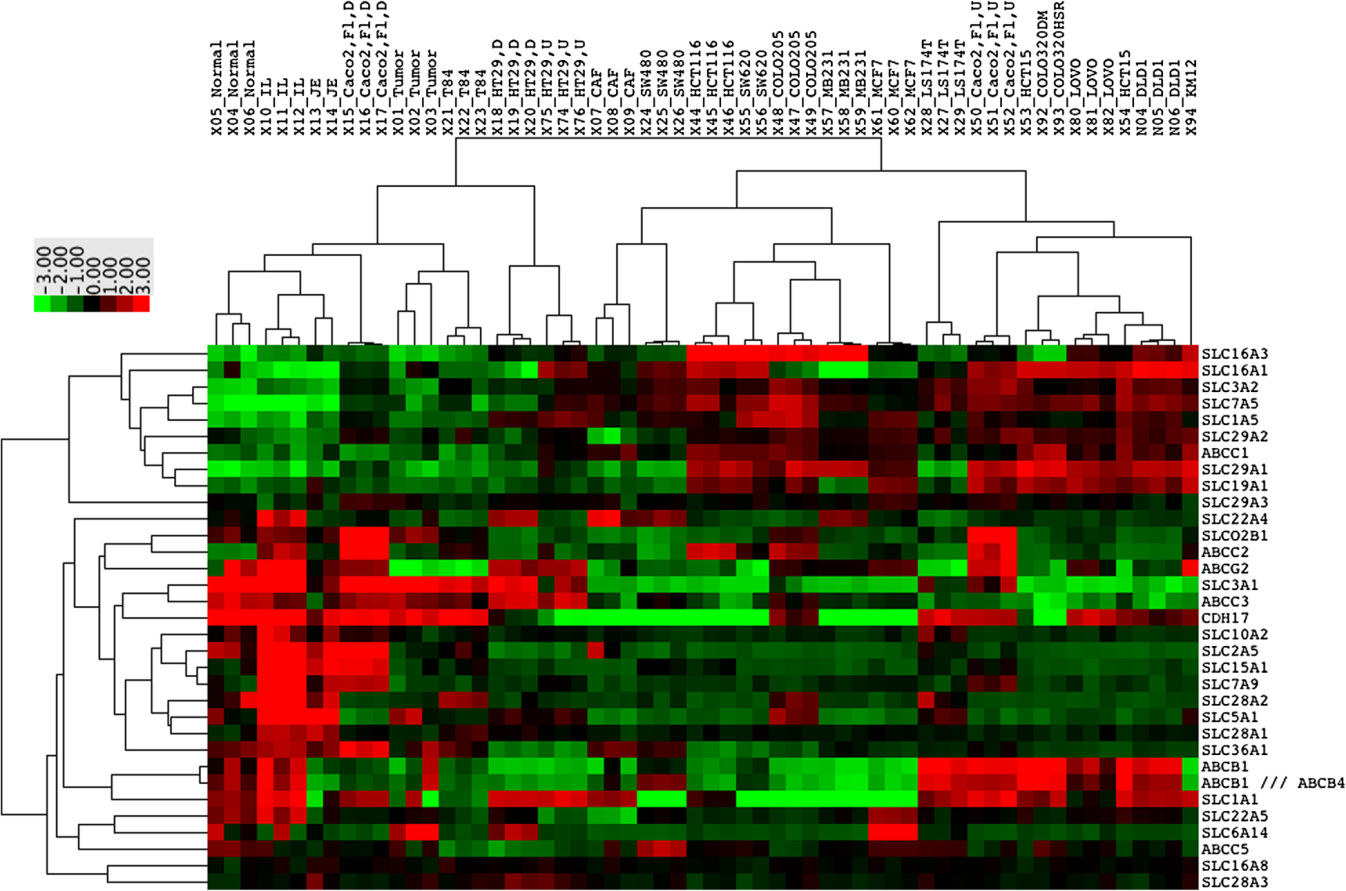
**Drug transporter profiles of small intestinal enterocytes most similar to Caco-2 cells.** Heatmap of the relative expression levels of transporters known to be relevant for oral drug absorption (Trans_BC). Tumor = laser dissected tumor cells, Normal = laser dissected colonocytes, JE, IL = laser dissected enterocytes of the jejunum or ileum, respectively, D = differentiated, U = undifferentiated, Fi = grown on filters, Fl = grown in flasks. Detailed description of samples in Additional file 5: Table S1.

Consequently, we made use of our objective approach to assess the suitability of the Caco-2 cells as model for oral drug absorption as compared to other CRC derived cell lines, taking inter-laboratory differences into account. As described above, PCA suggested that T84, HT29 and Caco-2 cells grown to confluency for 3 weeks were most similar to small intestinal enterocytes. Yet, when performing PCA filtering on solute carriers, transporters mostly responsible for active uptake, differentiated Caco-2 cells emerged as most akin to small intestinal enterocytes (Figure 4). When the analysis was extended to include expression of drug transporters known to be involved in uptake and secretion in the small intestine (Trans_BC [41]), differentiated Caco-2 cells clustered with normal colonocytes as well as small intestinal enterocytes (Figure 5). However, when focusing only on transporters potentially relevant for uptake (i.e. solute carriers expressed in plasma membrane according to Gene Ontology, SLC_PM), differentiated Caco-2 cells clustered only with small intestinal enterocytes (Additional file 2: Figure S1). When focusing on ABC transporters, differentiated Caco-2 cells clustered with HT29 and T84 cells (Additional file 3: Figure S2), suggesting that these cell lines could also serve as a model for active export. Focusing on the expression of SLCs specific for the small intestine (i.e. SLC_SI3: SLCs expressed ≥ two fold in small intestine versus normal pool samples), differentiated Caco-2 cells, however, ranked highest independent of culture conditions and origins (Table 1).

### Selection criteria for chemo-sensitivity studies

Recent reports suggest that chemotherapy may lead to a selection of tumor cells with EMT and stem cell properties and increased chemo-resistance [12]. For example, Gupta et al. [11] suggested that treatment with paclitaxel as compared to salinomycin significantly enriched breast cancer cells with EMT properties. Thus, we examined their data set (GEO, GSE17215) and found that our EMT signature was also significantly enriched when performing GSEA (p = 0.000, FDR = 0.000, normalized enrichment score = 2.01). Using the same approach, however, we could not observe any significant enrichment (i.e. p < 0.05) of this EMT signature in either oxaliplatin-treated HT29, DLD1 and LOVO cells (GEO, GSE10405), or methotrexate-treated HT29 and Caco-2 cells (GEO, GSE16648) as compared to untreated cells. Of note, however, that the stem cell signature HuISC was enriched in methotrexate treated Caco-2 cells (p = 0.000, FDR = 0.000, normalized enrichment score = 2.08).

To extend this analysis we addressed more comprehensively to what extent EMT, HuISC and WNT signatures correlated with GI50 values of 50 commonly used chemotherapeutics in the NCI60 cell line panel (http://dtp.nci.nih.gov/docs/cancer/cancer_data.html).

**Figure 6:**
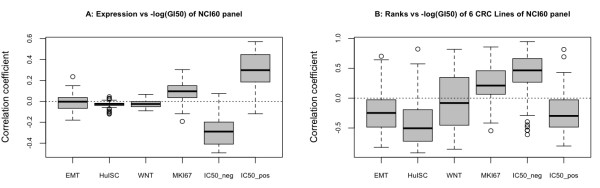
**Expression of EMT and HuISC gene sets do not correlate with GI50 values, but with a signature defined by mining NCI databases.** Boxplots of the median correlation coefficients between –log (GI50) values and (**A**) expression of various gene sets in the NCI60 cell line panel or (**B**) rank orders according to Table 1 of the six intestinal cell lines represented in the NCI60 panel (i.e. SW620, HCT116, HCT15, COLO205, KM12, HT29). Gene sets as described in Table 1.

Unexpectedly, no correlation between expression of EMT, HuISC and Wnt signatures and GI50 values was observed (Figure 6A), and furthermore, rank orders in terms of EMT, HuISC and Wnt signatures of 6 CRC cell lines comprised within the NCI60 panel (Table 1) did not reveal any positive correlation with GI50 values, yet even a tendency to a negative link (Figure 6B).

Most chemotherapeutics interfere with the cell cycle activities and accordingly cell proliferation has been associated with susceptibility to chemotherapy [42]. As expected, a positive correlation between expression of proliferation associated genes and GI50 values was observed across the NCI60 cell lines (Figure 6A). The CRC cell lines tested herein (Table 1) displayed no significant difference in proliferation (data not shown) and no link was observed between their rank orders (Table 1) and GI50 values (Figure 6B).

Finally, we scrutinized the NCI60 data sets for genes whose expression was either positively or negatively correlated with GI50 values of chemotherapeutics (i.e. for at least three compounds correlation coefficient > |0.5|). Clustering of the correlation coefficients revealed two distinct gene sets (Additional file 4: Figure S3) for which the median positive and negative correlation coefficients were determined (Figure 6A). Interestingly, a link could be observed between rank orders of the CRC cell lines (Table 1) and GI50 values (Figure 6B), suggesting that these new signatures might be worth pursuing for assessing chemo-sensitivity.

## Discussion

Selection criteria for cell-based models are often ill defined and arbitrary. Here we propose to make use of publicly available data generated by high throughput technologies to guide the choice of cell lines according to more objective criteria, such as gene expression signatures for selected traits. Focusing on CRC cell lines, we validated this approach for three different fields of application, i.e. the identification of cell lines most relevant for modeling and investigating tumor cell invasion, drug absorption and/or transport, and response to chemotherapy. Though we have focused on specific selection criteria, our strategy may be applied to any type of selection criteria (i.e. gene sets of different pathways and/or properties).

### Models to study tumor biology

In view of the heterogeneity of solid tumors [9,43] and the challenge to identify adequate cell-based models, we assessed to what extent comparative data mining for identifying tumor traits that overlap in tumor samples and cell lines might contribute to improving the relevance of cell-based models. We therefore ranked a panel of CRC cell lines using EMT and HuISC signatures to assess to what extent such signatures reflect invasive tumor behavior. Reports on in vivo studies with these cell lines indeed validate our ranking criteria. Thus, SW480, SW620, COLO320 and HCT116 cells, which all ranked high in our assessment for EMT and HuISC signatures, showed the highest propensity for local invasion upon orthotopic grafting [6,8], whereas HT29, COLO205 and DLD1, which ranked lower for these traits, showed less aggressive behavior. De Vries and co-workers observed that SW620 cells were indeed strongly invasive in vitro, in vivo, however, they proved less invasive than HT29 and LS174T cells [44]. Collectively, unbiased data mining allowed identifying cell lines with prominent mesenchymal traits (e.g. SW480), which display migratory properties and represent an invasive phenotype, whereas HT29, the most frequently used CRC model line, and T84 cells, which both ranked low for these traits came closest to gene expression profiles of bulk tumor tissue cells (Figure 1 and Table 1).

### Caco-2 cells, a model of choice for drug absorption

Screening the literature with key words such as “absorption” and “transporters” revealed that, within the CRC cell line panel assessed herein, Caco-2 is the most widely used intestinal cell line for absorption-related studies [45-47]. Comparing transporter gene expression profiles revealed that differentiated Caco-2 cells indeed most accurately recapitulate those of normal enterocytes. Similar to Hayeshi et al. [37], we observed that culture conditions influence the expression pattern of selected transporters. Based on our ranking method we show that Caco-2 cells grown for three weeks, independently of their origin and culture conditions, best match the genomic profiles of small intestinal enterocytes.

### Chemo-sensitivity

Selection of tumor cells with EMT and stem cell properties and higher chemo-resistance has recently emerged as a novel mechanism underlying therapy-resistance [10-12,48,49]. Vast databases with GI50 values, a standard to assess drug sensitivity [15,16], are available for the intestinal CRC lines HT29, COLO205, KM12, SW620, HCT116, HCC_2998 and HCT15 through the NCI database (http://dtp.nci.nih.gov/docs/cancer/cancer_data.html). We therefore probed these data sets for a correlation between EMT and CSC signatures and chemoresistance. Using the EMT signature defined herein, we could indeed confirm such a correlation in the breast cancer data set of Gupta et al. [11]. However, we could not observe any significant association of EMT-related genes with the GI50 values of CRC cell lines (Figure 6). Interestingly, novel chemoresistance-associated signatures could be identified; indicating that in CRC cell lines expression of EMT and CSC signatures are not dominant factors in the response to therapy. Cell line- or treatment-specific effects may account for this discrepancy. It is of note that Gupta and co-authors compared paclitaxel vs. salinomycin-treated cells, while the other data were based on treatment versus non-treatment comparisons.

A correlation between drug resistance and EMT features has, however, been observed using CRC biopsies [13,14]. As these may contain variable amounts of stroma and as stroma content may influence response to therapy [50], these data are not directly comparable to GI50 values of CRC cell lines.

## Conclusions

We have presented a straightforward strategy to exploit publicly available gene expression data to guide the choice of cell-based models. While this approach does not overcome the major limitations of such models, introducing a rank order of selected features may allow selecting model cell lines that are more adapted and pertinent to the addressed biological question. In summary, we conclude that SW480 represent an EMT-high, migratory phenotype and scored highest in terms of signatures associated to worse overall survival and higher risk of recurrence based on patient derived databases, therefore a good model to study invasive tumor cells. On the other hand, differentiated HT29 and T84 cells showed gene expression patterns closest to tumor bulk derived cells. Regarding drug absorption, we confirmed that differentiated Caco-2 cells are the model of choice for active uptake studies in the small intestine.

## Methods

### Cell culture

Cell culture media were as follows: SW480, RPMI 1640, 10% fetal calf serum (FCS); Caco-2, Dulbecco’s modified Eagle essential medium (DMEM) Glutamax, 10% FCS, 1% non-essential amino acids; HT29, LS174T, DMEM Glutamax, 10% FCS; T84, RPMI 1640, 10% FCS, 2 mM glutamine. Primary fibroblasts, DMEM Glutamax, 10% FCS, 100 U/ml penicillin, 100 μg/ml streptomycin, pt?>0.5 mL gentamycin (50 mg/mL). All cell lines were cultured at 37°C and 5% CO_2_ (Samples 15–29 see Additional file 5: Table S1). CDX2-shCaco-2 cells were maintained under puromycin selection (2 ug/mL) (Samples 86–88 see Additional file 5: Table S1). RNA was extracted using Nucleo-Spin RNA-extraction kit from Machery-Nagel (Oensingen, Switzerland). CacoReady^TM^ kit cells (Readycell, Barcelona, Spain) were cultured on 24-transwells filters for either 14 days in DMEM low glucose containing 10% FCS, 1% L-glutamine and 100 U/ml penicillin, 100 μg/ml streptomycin, then 4 days of culture in semi-solid shipping media and again 3 days in culture media (Readycell’s patented technology) (Samples 54–56 see Additional file 5: Table S1), or for 3 weeks in the absence of the semi-solid shipping media (Samples 57–59 see Additional file 5: Table S1). Differentiated Caco-2LD cells were grown at low density and then differentiated on 0.4 μm PET filters as described earlier [51]

### Laser dissection microscopy

Immediately after resection of human tissue, samples were put on ice and sections of one cm length of small intestine and colon (tumor regions and adjacent normal epithelium), respectively, were cut, washed in chilled PBS, embedded in OCT and frozen immediately. One section of the ileum und two of the jejunum were first stored in RNAlater at 4°C for shipping, then rinsed with chilled PBS and embedded in OCT and frozen immediately. 12 μm frozen sections were cut and mounted on Leica or Zeiss membranes for dissecting microscopy (Leica Microsystems, Germany and PALM Microbeam, Germany), fixed in 96% ethanol for 30 s and colored for an equivalent time with hematoxylin/eosin solution, respectively. Membranes were then rinsed in water for 30 s, transferred for 10 s to 70% ethanol, followed by 96% ethanol and air-dried. Frozen samples were processed using a laser dissecting microscope coupled to a CCD camera (Leica Microsystems, Germany and PALM Microbeam, Germany). For each patient sample dissected cells were pooled in a tube cap containing 20 μL RNA lysis buffer. Collection of human intestinal samples was approved by the corresponding local ethic commission (Samples 1–6, 10–14 see Additional file 5: Table S1).

### Gene expression using GeneChip® human genome U133 plus 2.0

Total RNA was extracted using the total RNA extraction Nucleospin II kit by Machery-Nagel (Oensingen, Switzerland). The quality and quantity of all RNA samples was examined by the Agilent 2100 Bioanalyzer (Agilent Biotechnologies, Germany) and by a NanoDrop (Witec AG, Switzerland), respectively. RINs for cell lines were between 9 and 10, for LDM material between 5 and 7 except for two small intestinal samples. 100 ng of total RNA were used as the starting material for all individual samples. Labeling and fragmentation of cRNA, array hybridization and scanning was performed according to the protocol by Affymetrix. Details see Additional file 6. Cell lines were measured in triplicates with different passage numbers. Normal tumor intestinal human tissue was obtained from different individuals. The complete data set is publicly available at http://www.ncbi.nlm.nih.gov/geo/ through the accession number GSE30292.

### Migration and proliferation assay

Chemotaxis was assayed in 48-well Boyden microchambers (Neuro Probe, Cabin John, MD, USA). Cells were cultured in serum-free media overnight. For the migration studies, chemotaxis buffer containing 30% serum was placed in the lower wells, and 10^4^ cells, suspended in chemotaxis buffer (culture media with 1% BSA), in the upper wells. A PVPF membrane (Poretics 25 × 80 mm) with 8 μm pores was coated with rat collagen type I. After 15 h of incubation the membrane was removed, washed on the upper side with PBS, fixed and stained with Diff-Quick staining kit. Migrated cells were counted at 1000-fold magnification in five randomly selected fields. Values are given as average cell count of five high-powered fields (5 HPF). Proliferation was assessed using an MTT assay (Sigma-Aldrich, Switzerland) over five days. Briefly, 3000 cells were seeded in five replicates per day, per well in a 96-well plate. At each time point 10 μL MTT working solution was added to each well. Then the plate was incubated for 3 h at 37°C and the cells lysed with lysis buffer overnight. Optical density was measured at 540 nm. ANOVA was done for migration data (p value ≤ 0.05).

### Public data

Publicly available data was obtained at http://www.ncbi.nlm.nih.gov/geo/ through the accession numbers GSE2361, GSE2361, GSE7303 (Pool normal; colon GSM175905, small intestine GSM175908, lung GSM176012, breast GSM175792, prostate GSM175923, uterus GSM175945, kidney GSM175911), GSE2109 (Pool tumor; colon GSM38055, small intestine GSM38068, lung GSM38051, breast GSM38051, prostate GSM38053, uterus GSM38052, kidney GSM38073), GSE10843, GSE13059, GSE16648, GSE22572. MB231 and MCF7 cell lines (GSE10890) were included in the analysis as controls for cells with strong EMT properties and with weak, respectively. Expression data for NCI60 cell line panel was obtained through http://discover.nci.nih.gov/cellminer/[52] and GI50 data at http://dtp.nci.nih.gov/docs/cancer/cancer_data.html.

### Expression data analysis

Robust multi-array averaging (RMA) and quantile normalization were used to quantify gene expression. Significant differences were identified applying a Bayesian approach using the limma package (R 2.12.0, Bioconductor 2.7). A threshold of an adjusted p value ≤ 0.05 was used to identify significant changes if not indicated otherwise. Gene set enrichment analysis (GSEA) was carried out according to Subramanian et al. [53] and p values were computed using a bootstrap distribution created by resampling gene sets of the same cardinality. See details in supplementary data section “expression data analysis” and Additional file 1: Table S2 regarding data sets. Principle component analysis was performed using the affy package (R, Bioconductor). Correlation coefficients between –log(GI50) of selected drugs (see Additional file 6) and normalized expression values were calculated using R. Heatmaps were generated using Cluster 3.0 and TreeView (http://rana.lbl.gov/EisenSoftware.htm) [54]. Centered correlation or Spearman rank correlation and average linkage clustering, respectively, were used for similarity measurement and clustering. Effects of signatures on overall and recurrence free survival were studied using the Kaplan-Meier survival curves and log rank tests in three different publicly available data sets (GSE12945, GSE14333, GSE17537) using the survival package in the statistical software R version 2.13.0. Patient samples were divided into two groups splitting at the average score.

### Ranking according to signature strength

Gene expression data was filtered by selecting only one probe set, the one with the highest standard deviation across all samples, per gene. For each gene of a given gene set the relative expression (i.e. gene-wise zero-centering of expression values) across all samples was determined. The score of a gene set was calculated as the average of the expression values of the genes in the set. The cell lines were then ranked according to this score. See details in supplementary data section “expression data analysis” and Additional file 1: Table S2 regarding data sets.

### Drug sensitivity analysis

Correlation coefficients between –log(GI50) and log2 gene expression values were calculated for a panel of 50 chemotherapeutics (CTX, list of drugs see Additional file 7: Figure S4) and a panel of drugs (NSC) for which for all 59 cell lines GI50 values were available and SD across 59 lines of –log(GI50) was > 0.

## Competing interests

The authors declare that they have no competing interests.

## Authors’ contributions

Data acquisition: JC, SE, MN, PA, SB, MV, AF, SS, JH; data analysis: PA, JC, SE, TS, MD, MA; patient samples: MR, HB, PS, JC, PA; study design, funding, drafting of ms: PA, MA; supervision: PA. All authors read and approved the final manuscript.

## Supplementary Material

Additional file 1 Table S2.Gene sets used for data analysis Click here for file

Additional file 2 **Figure S1. Solute carriers of plasma membrane profiles of small intestinal enterocytes most similar to Caco-2 cells.** Heatmap of relative expression levels of solute carriers of the plasma membrane. Click here for file

Additional file 3 **Figure S2. Differentiated Caco-2, HT29 and T84 cell lines most similar to enterocytes with respect to expression ABC transporters.** Heatmap of the relative expression levels of ABC transporters. Click here for file

Additional file 4 **Figure S3. Two distinct groups of genes with respect to expected chemosensitivity.** Heatmap of correlation coefficients –log(GI50) of 50 chemotherapeutics and expression values across the NCI60 cell line panel. Click here for file

Additional file 5 Table 1S. List of expression files used for microarray analysis and corresponding accession numbers at Gene Expression Omnibus. Click here for file

Additional file 6 **Supplementary information for methods and additional results[**29,30,41,55-58**]** Click here for file

Additional file 7 **Figure S4. Enrichment of small intestinal signature in Caco-2 cells upon differentiation.** Heatmap representing enrichment scores obtained by gene set enrichment analysis of the selected panel of gene sets. Genes in the various cell lines were ranked according to their relative average expression (n = 2-3) in the given cell line compared to a panel of healthy epithelial tissues. Details see Additional file 6, material and methods. Click here for file
