# Supplementary figures and images for: Defining new criteria for selection of cell-based intestinal models using publicly available databases
Source: BMC Genomics. 2012 Jun 22;13:274. doi: 10.1186/1471-2164-13-274 (PMC3412164; doi:10.1186/1471-2164-13-274)

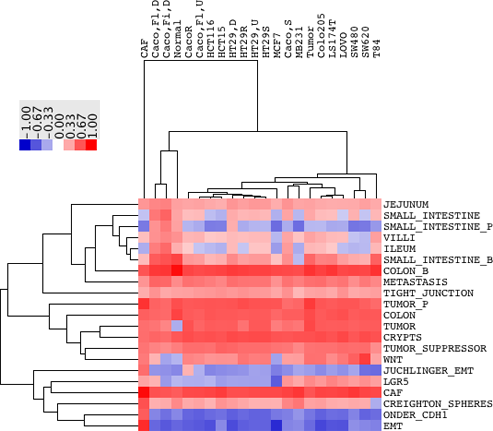

Supplement: Additional file 4 — Figure S3. Two distinct groups of genes with respect to expected chemosensitivity. Heatmap of correlation coefficients –log(GI50) of 50 chemotherapeutics and expression values across the NCI60 cell line panel. [file 1471-2164-13-274-S4.tiff]
