# Supplementary material for: Defining new criteria for selection of cell-based intestinal models using publicly available databases
Source: BMC Genomics. 2012 Jun 22;13:274. doi: 10.1186/1471-2164-13-274 (PMC3412164; doi:10.1186/1471-2164-13-274)
Supplement: Additional file 5 — Table 1S. List of expression files used for microarray analysis and corresponding accession numbers at Gene Expression Omnibus. [file 1471-2164-13-274-S5.pdf]

Table 1S: List of expression files used for microarray analysis and corresponding accession numbers at Gene Expression Omnibus.

Sample Nr.	Chip Names	GSE Reference	Sample Names	Names Figure 1 and 4
1	01_E3.CEL	GSE30292	Tumor	E=tumor cells
2	02_E8.CEL	GSE30292	Tumor	E=tumor cells
3	03_E9.CEL	GSE30292	Tumor	E=tumor cells
4	04_N3.CEL	GSE30292	Normal	N= healthy colonocytes
5	05_N8.CEL	GSE30292	Normal	N= healthy colonocytes
6	06_N14.CEL	GSE30292	Normal	N= healthy colonocytes
7	07_CAF8.CEL	GSE30292	CAF	F=cancer-associated fibroblasts
8	08_CAF14.CEL	GSE30292	CAF	F=cancer-associated fibroblasts
9	09_CAF17.CEL	GSE30292	CAF	F=cancer-associated fibroblasts
10	10_IL_Biopredict.CEL	GSE30292	IL	I=small intestinal enterocytes
11	11_IL_PAT31.CEL	GSE30292	IL	I=small intestinal enterocytes
12	12_IL_Spain.CEL	GSE30292	IL	I=small intestinal enterocytes
13	13_JE_PAT25.CEL	GSE30292	JE	I=small intestinal enterocytes
14	14_JE_PAT26.CEL	GSE30292	JE	I=small intestinal enterocytes
15	15_Caco2.CEL	GSE30292	Caco,Fl,D	Caco-2=C (differentiated)
16	16_Caco2.CEL	GSE30292	Caco,Fl,D	Caco-2=C (differentiated)
17	17_Caco2.CEL	GSE30292	Caco,Fl,D	Caco-2=C (differentiated)
18	18_HT29.CEL	GSE30292	HT29,D	HT29=H (differentiated)
19	19_HT29.CEL	GSE30292	HT29,D	HT29=H (differentiated)
20	20_HT29.CEL	GSE30292	HT29,D	HT29=H (differentiated)
21	21_T84.CEL	GSE30292	T84	T84=T
22	22_T84.CEL	GSE30292	T84	T84=T
23	23_T84.CEL	GSE30292	T84	T84=T
24	24_SW480.CEL	GSE30292	SW480	SW480=S
25	25_SW480.CEL	GSE30292	SW480	SW480=S
26	26_SW480.CEL	GSE30292	SW480	SW480=S
27	27_LS174T.CEL	GSE30292	LS174T	LS174T=L
28	28_LS174T.CEL	GSE30292	LS174T	LS174T=L
29	29_LS174T.CEL	GSE30292	LS174T	LS174T=L
30	44_HCT116_GSM274713.CEL	GSE10843	HCT116	HCT116=6
31	45_HCT116_GSM274714.CEL	GSE10843	HCT116	HCT116=6
32	46_HCT116_GSM274726.CEL	GSE10843	HCT116	HCT116=6
33	47_Colo205_GSM274717.CEL	GSE10843	COLO205	COLO205=O
34	48_Colo205_GSM274718.CEL	GSE10843	COLO205	COLO205=O
35	49_Colo205_GSM274728.CEL	GSE10843	COLO205	COLO205=O
36	50_Caco2_GSM274711.CEL	GSE10843	Caco,Fl,U	G=Caco-2 (undifferentiated)
37	51_Caco2_GSM274712.CEL	GSE10843	Caco,Fl,U	G=Caco-2 (undifferentiated)
38	52_Caco2_GSM274725.CEL	GSE10843	Caco,Fl,U	G=Caco-2 (undifferentiated)
39	53_HCT15_GSM274763.CEL	GSE10843	HCT15	HCT15=5
40	54_HCT15_GSM274764.CEL	GSE10843	HCT15	HCT15=5
41	55_SW620_GSM274773.CEL	GSE10843	SW620	SW620=W
42	56_SW620_GSM274774.CEL	GSE10843	SW620	SW620=W
43	57_MB231_GSM276040.CEL	GSE10890	MB231	MB231=M
44	58_MB231_GSM276041.CEL	GSE10890	MB231	MB231=M
45	59_MB231_GSM276042.CEL	GSE10890	MB231	MB231=M
46	60_MCF7_GSM276049.CEL	GSE10890	MCF7	MCF7=7
47	61_MCF7_GSM276050.CEL	GSE10890	MCF7	MCF7=7
48	62_MCF7_GSM276051.CEL	GSE10890	MCF7	MCF7=7
49	63_cacoF.CEL	GSE30292	Caco,Fi,D	
50	64_cacoF.CEL	GSE30292	Caco,Fi,D	
51	65_cacoF.CEL	GSE30292	Caco,Fi,D	
52	66_caco_JU.CEL	GSE30292		
53	67_caco_JD.CEL	GSE30292		
54	68_cacoB.CEL	GSE30292		
55	69_cacoB.CEL	GSE30292		
56	70_cacoB.CEL	GSE30292		
57	71_cacoR.CEL	GSE30292	CacoReady	
58	72_cacoR.CEL	GSE30292	CacoReady	
59	73_cacoR.CEL	GSE30292	CacoReady	
60	74_HT29_GSM327229.CEL	GSE13059	HT29,U	HT29=9
61	75_HT29_GSM327230.CEL	GSE13059	HT29,U	HT29=9
62	76_HT29_GSM327231.CEL	GSE13059	HT29,U	HT29=9
63	77_CacoP2_GSM402370.CEL	GSE16648	Caco,S	
64	78_CacoP2_GSM402373.CEL	GSE16648	Caco,S	
65	79_CacoP2_GSM402375.CEL	GSE16648	Caco,S	
66	80_Lovo_GSM274719.CEL	GSE10843	LOVO	LOVO=V
67	81_Lovo_GSM274720.CEL	GSE10843	LOVO	LOVO=V
68	82_Lovo_GSM274729.CEL	GSE10843	LOVO	LOVO=V
69	83_HT29_GSM288491.CEL	GSE16648	HT29,S	
70	84_HT29_GSM288497.CEL	GSE16648	HT29,S	
71	85_HT29_GSM288499.CEL	GSE16648	HT29,S	
72	86_HT29_GSM288501.CEL	GSE16648	HT29,R	
73	87_HT29_GSM288502.CEL	GSE16648	HT29,R	
74	88_HT29_GSM288536.CEL	GSE16648	HT29,R	
75	89_Caco2_GSM402378.CEL	GSE16648	Caco,R	
76	90_Caco2_GSM402380.CEL	GSE16648	Caco,R	
77	91_Caco2_GSM402383.CEL	GSE16648	Caco,R	
78	92_COLO320DM_GSM274777.CEl	GSE10843	COLO320	COLO320=3
79	93_COLO320HSR_GSM274778.CE	GSE10843	COLO320	COLO320=3
80	94_KM12_GSM274781.CEL	GSE10843	KM12	KM12=K
81	95_CDX22_GSM564445.CEL	GSE22572	Caco_CDX2,D	
82	96_CDX22b_GSM564448.CEL	GSE22572	Caco_CDX2,D	
83	97_CDX24_GSM564446.CEL	GSE22572	Caco_CDX2,D	
84	98_GFP1_GSM564444.CEL	GSE22572		
85	99_GFP2_GSM564447.CEL	GSE22572		
86	N01_CacoCDX21.CEL	GSE30292	Caco_CDX2,U	
87	N02_CacoCDX22.CEL	GSE30292	Caco_CDX2,U	
88	N03_CacoGFP.CEL	GSE30292		
89	N04_DLD1_GSM274715.CEL	GSE10843	DLD1	DLD1=D
90	N05_DLD1_GSM274716.CEL	GSE10843	DLD1	DLD1=D
91	N06_DLD1_GSM274727.CEL	GSE10843	DLD1	DLD1=D
