# Supplementary material for: Defining new criteria for selection of cell-based intestinal models using publicly available databases
Source: BMC Genomics. 2012 Jun 22;13:274. doi: 10.1186/1471-2164-13-274 (PMC3412164; doi:10.1186/1471-2164-13-274)
Supplement: Additional file 6 — Supplementary information for methods and additional results[29,30,41,55-58] [file 1471-2164-13-274-S6.docx]

**Supplementary Data**

**Supplementary Data: Material and Methods**

**Transfections and transductions**: Short hairpin RNAs (shRNA) targeting CXD2 were individually cloned into plko.1-puromycin vector purchased from Open Biosystem (SHGLY-NM_001265, Sigma-Aldrich, Switzerland). Briefly, lentiviral stocks were prepared using HEK293FT as packaging cells. Nearly confluent cells were transfected by the standard lipofectamine method with plasmids mix (5 ug of pLP1, 2.5 ug pLP2, 3 ug pLP/VSVG plasmids and 10 ug of each plko.1-containing CDX2 shRNA inserts or pLentiV5-GFP). HEK293FT cells were grown in DMEM media supplemented with 10% FBS, non-essential amino acids, glutamine and Na-pyruvate. For transfection, plasmid mix was diluted in 400 uL OPTIMEM, at the same time 45 uL lipofectamine2000 was diluted in 400 uL OPTIMEM. Following 5 min incubation at room temperature, the two solutions were combined and the mixture was added to the cells after 20 minutes of incubation at room temperature. The day after, the transfection mix was removed and fresh medium was added. After 48 hours the medium containing lentivirus was collected, stored at -80 °C (48h virus medium) and new fresh medium was added to the HEK293FT cells. After 24 hours the medium containing lentivirus was collected and stored at -80 °C (72h virus medium). One million of growing Caco-2 cells were harvested with trypsin solution, resuspended in 1 mL of 48h-virus-medium plus 4 uL polybrene and seeded on p32 tissue culture dish. One hour after seeding, 1 mL of complete medium (DMEM w/o antibiotics) with 4 uL polybrene were added. Cells were cultured for 2 days without changing the medium. After 2 days post infection, cells were harvested and seeded on p90 tissue culture dishes. From this step on, shCaco-2 cells were maintained under puromycin selection (2 ug/mL). The protein knockdown efficiency protein measured by Western blots was in the range of 60-80 % in 5-day-cultured and 30-67 % in 21-day-cultured Caco-2 cells (data not shown). Earlier studies have shown that total CDX2 removal resulted in cell death and viable cells tolerated a reduction at the protein level only to 70% [55].

**Expression data analysis:** Gene sets tested were defined as follows:

A) Gene sets used for Table 1:

1. **WNT** (direct Wnt targets) and **EMT**: same gene sets as described in [29];
2. **HuISC**: set of genes, recently being described as “humanized intestinal stem cell signature” (HuISC), which identified recurrent CRC [30];
3. **Trans_BC**: Drug transporters known to be involved in uptake and secretion in the small intestine [41];
4. **SLC_PM, SLC_SI3**: Solute carriers were defined as the collection of members of the SLC family based on their official gene symbols (http://www.genenames.org/genefamilies): SLC_PM, solute carriers expressed in plasma membrane according to Gene Ontology, SLC_SI3: SLCs expressed ≥ two fold in small intestine versus normal pool samples;
5. **ABC**: ABC transporters were defined as the collection of members of ABC superfamily based on their official gene symbols (http://www.genenames.org/genefamilies);
6. **MKI67_CO**: Genes which correlate with MKI67 expression (t ≥ 15) in colon cancer biopsy samples of the expO data set (http://www.intgen.org/expo/).
7. **IC50_pos and IC50_neg**: Genes for which absolute correlation coefficients |> 0.5| between log2 gene expression values across 60 cell lines and –log(GI50) of at least 3 chemotherapeutics was measured. Positive and negative list was according to cluster separation shown in Figure 3S.

B) Other gene sets:

1. **SMALL_INTESTINE**: genes overexpressed in samples 10-14 Table 1S vs. samples 4-6, adjusted p value < 0.01, log2 fold change > 2;
2. **JEJUNUM**: genes which are overexpressed in samples 13-14 Table 1S vs. samples 4-6 (adjusted p value < 0.01, log2 fold change > 2;
3. **ILEUM**: genes overexpressed in samples 10-12 Table 1S vs. samples 4-6 (adjusted p value < 0.01, log2 fold change > 2);
4. **SMALL_INTESTINE_P**: genes overexpressed in small intestinal sample (i.e. GSE2361, GSM44679) vs. colon sample (i.e. GSE2361, GSM44680, log 2 fold change > 1.5);
5. **COLON**: genes overexpressed in samples 10-12 Table 1S vs. samples 4-6 (adjusted p value < 0.01, log2 fold change < -2);
6. **TUMOR**: Genes overexpressed in samples 1-3 Table 1S vs. samples 4-6 (adjusted p value < 0.05, log2 fold change > 2);
7. **TUMOR_P**: genes overexpressed in pool of three colon tumor samples (GSE2109; GSM38074, GSM38075, GSM38077) vs. normal pool samples (log2 fold change >2);
8. **VILLI**: see [56];
9. **CRYPTS**: see [56];
10. **LGR5**: see [29, 57];
11. **ONDER_CDH1**: genes overexpressed in shCDH1 treated cells vs. wild-type cells (GSE9691, adjusted p value < 0.001, log2 fold change > 2);
12. **CREIGHTON_SPHERES**: genes overexpressed in spheres vs. bulk tumor samples (GSE7515, adjusted p value < 0.001, log2 fold change > 2);
13. **METASTASIS**: see [58];
14. **SMALL_INTESTINE_B**: genes overexpressed in samples 10-14 Table 1S vs. normal pool samples (adjusted p value < 0.01, log2 fold change > 3);
15. **COLON_B**: Genes overexpressed in samples 4-6 Table 1S vs. normal pool samples (adjusted p value < 0.001, log2 fold change > 3),
16. **CAF**: Genes which are overexpressed in CAFs (i.e. samples 7-9 Table 1S vs. normal pool samples, adjusted p value <0.001, log2 fold change > 3);
17. **JUCHLINGER_EMT, TUMOR_SUPPRESSORS,** and **TIGHT_JUNCTION**: see MiSigDB (http://www.broadinstitute.org/gsea);

**Definition of significant differences:** For genomics data of own LDM material with known high RNA quality (i.e. gene sets #8, 9, 10, 12) an adj. p <0.01 and log2FC cutoff 2 were applied to define significant differences, except for gene set 13 in order to adjust for similar gene set sizes. Regarding analysis of public data (i.e. gene sets 18, 19, 21, 22, 23) more stringent conditions were applied by decreasing the cut-off of the p values and/or increasing cut-off of relevant fold changes.

See Table 2S for detailed lists of all genes sets.

**Supplementary Data: Results**

**Cell line specific features.** To elucidate which functional or physiological features are specific for the various cell lines, gene set enrichment analysis (GSEA) of various gene sets associated to tumor progression, EMT and differentiation was performed. The genes were ranked according to their expression in the various cell lines compared to a common reference consisting of a pool of seven normal epithelial tissue samples (i.e. colon, small intestine, breast, lung, prostate, uterus, kidney). The enrichment scores are shown in Figure 4S. Differentiated Caco-2 cells showed the strongest enrichment of “small intestinal signatures”. This includes genes expressed at higher levels in the small intestine compared to colon. While the “villi signature” was similarly enriched in Caco-2, HT29, and T84 cells, the “Wnt signature” was strongest in the SW cell lines. Signatures related to mesenchymal characteristics (i.e. JUCHLINGER_EMT, Onder_CDH1 and EMT) were all negatively enriched except in CAFs, the positive control. The SW480 and MB231, a breast cancer cell line with strong fibroblast-like morphology derived from adenocarcinoma, showed the weakest negative enrichment. Signatures related to stemness (LGR5, CREIGHTON_SPHERES), metastasis (METASTASIS), tumor features (TUMOR, TUMOR_P, TUMOR-SUPPRESSORS), colon (COLON, COLON_B) and crypts (CRYPTS) were not specifically enriched in any cell line.

**Supplementary Data: Tables**

Table 1S: **List of expression files used for microarray analysis and corresponding accession numbers at Gene Expression Omnibus.**

Table 2S: **Gene sets used for data analysis**

**Supplementary Data: Figure caption legend**

Figure 1S: **Solute carriers of plasma membrane profiles of small intestinal enterocytes most similar to Caco-2 cells**. Heatmap of relative expression levels of solute carriers of the plasma membrane.

Figure 2S: **Differentiated Caco-2, HT29 and T84 cell lines most similar to enterocytes with respect to expression ABC transporters**. Heatmap of the relative expression levels of ABC transporters.

Figure 3S: **Two distinct groups of genes with respect to expected chemosensitivity**. Heatmap of correlation coefficients –log(GI50) of 50 chemotherapeutics and expression values across the NCI60 cell line panel.

Figure 4S: **Enrichment of small intestinal signature in Caco-2 cells upon differentiation**. Heatmap representing enrichment scores obtained by gene set enrichment analysis of the selected panel of gene sets. Genes in the various cell lines were ranked according to their relative average expression (n=2-3) in the given cell line compared to a panel of healthy epithelial tissues. Details see supplementary data, material and methods.
